# Beyond Fracture Probability: Communicating the Full Consequences of Fracture and Contextualization

**DOI:** 10.1007/s11914-026-00974-1

**Published:** 2026-06-30

**Authors:** Tuan V. Nguyen

**Affiliations:** 1 School of Population Health, UNSW Medicine, UNSW Sydney, Sydney, Australia; 2 School of Biomedical Engineering, University of Technology, Sydney (UTS), Broadway, Ultimo, Sydney, NSW 2007 Australia

**Keywords:** Osteoporosis, Fracture, Probability, Risk communication, Skeletal age, FRAX, Garvan fracture risk calculator, Qfracture, BONEcheck

## Abstract

**Purpose of Review:**

Fracture risk calculators such as FRAX, the Garvan Fracture Risk Calculator, and QFracture are now embedded in clinical guidelines, yet they communicate only a single numerical probability. This review examines whether current tools adequately meet patients' informational needs and identifies critical gaps in fracture risk communication.

**Recent Findings:**

Most patients want fracture risk information, but only approximately half actually receive it. Research consistently shows that patients globally prefer visual over numerical formats, yet existing tools do not communicate the consequences of fracture, including mortality, subsequent fracture risk, and loss of independence, nor do they contextualise risk within available treatment options. Three critical gaps are identified: the consequence gap (what fracture means for survival and function), the controllability gap (how treatment modifies risk), and the format gap (how risk is presented and understood).

**Summary:**

Moving fracture risk communication beyond a single number requires integrating consequence, context, and format. The digital platform BONEcheck addresses these gaps by incorporating mortality risk, refracture risk, skeletal age, treatment contextualisation, and multi-format presentation including icon arrays. Future research, clinical practice, and guideline development should prioritise a more complete, actionable, and patient-centred approach to fracture risk communication.

## Introduction

Over the past two decades, considerable effort has been devoted to identifying individuals at high fracture risk before they fracture. Tools such as FRAX [1], the Garvan Fracture Risk Calculator [2, 3], and QFracture [4] estimate 5- or 10-year fracture probability based on clinical risk factors, with or without bone mineral density (BMD). FRAX, first released in 2008, calculates 10-year probabilities of major osteoporotic fracture and hip fracture using age, sex, BMI, and seven dichotomous clinical risk factors (prior fracture, parental hip fracture, smoking, glucocorticoid use, excess alcohol, rheumatoid arthritis, secondary osteoporosis), with optional femoral neck BMD [1]. The Garvan Fracture Risk Calculator, first released in 2007 and developed from the Dubbo Osteoporosis Epidemiology Study in Australia, estimates 5- and 10-year fracture risk incorporating falls history and the number of prior fractures which are not quantitatively captured by FRAX [2, 3]. QFracture, developed from a large UK primary care database, provides 10-year fracture risk using a broader range of risk factors including falls, chronic conditions, and medications [4]. FRAX alone has been accessed over 4.3 million times in the United States and is incorporated into more than 100 international guidelines [5]. These tools represent a genuine advance in osteoporosis management.

Yet despite the widespread availability of fracture risk assessment tools and effective treatments, a persistent “treatment gap” remains: a large proportion of patients who have sustained a fracture or who are at high risk of fracture do not receive appropriate treatment [6, 7]. The problem of ineffective risk communication in osteoporosis management, and its direct role in perpetuating the treatment gap, was identified more than 10 years ago [8]. A fracture probability, however accurate, is only useful if it is understood by the patient, perceived as personally relevant, and accompanied by actionable information about what can be done.

Here, I argue that current fracture risk communication suffers from three critical gaps: a consequence gap (tools report fracture probability but not what fracture means for mortality, refracture, and independence), a controllability gap (tools report risk but not what treatment can do about it), and a format gap (tools present risk as a single numerical percentage rather than in formats shown to improve comprehension). I review the current practice and the relevant research literature, and we discuss how the digital tool BONEcheck (bonecheck.org) [9] addresses all three gaps within a single clinically feasible platform.

## Current Practice in Fracture Risk Communication

Currently, all fracture risk assessment tools produce the same type of output: a single probability expressed as a percentage, for example, “*Your 10-year risk of a major osteoporotic fracture is X%.*” This figure is delivered with little to no interpretation, leaving out any context about what such a fracture would actually mean for the patient’s quality of life or health outcomes, and providing no information about how treatment options might reduce that risk.

While the effectiveness of fracture risk assessment tools depends critically on how results are communicated to patients [8], this area has received far less attention than in cardiovascular medicine. Major clinical guidelines from organizations such as the International Osteoporosis Foundation (IOF), the American Association of Clinical Endocrinology (AACE), the National Osteoporosis Guideline Group (NOGG), and the Endocrine Society focus primarily on treatment thresholds: at what fracture probability should pharmacotherapy be initiated [10–12]. Apart from NOGG which includes brief recommendations on communicating fracture risk, other guidelines provide detailed recommendations on when to treat but offer little guidance on how to communicate risk to patients. By contrast, guidelines in cardiovascular risk management have increasingly incorporated recommendations for risk communication formats, including the use of absolute risk, visual aids, and age-based framing [13].

Many clinicians use fracture risk scores primarily for threshold-based treatment decisions (i.e., determining whether a patient’s risk exceeds a guideline-specified threshold for pharmacotherapy) rather than as a basis for shared decision-making with patients [14, 15]. In cardiovascular disease, a qualitative study of general practitioners found that many did not share the numerical output with patients, or shared it without interpretation, partly because they found the numbers difficult to explain and partly because they were uncertain whether patients would understand or benefit from hearing them [16].

The RICO (Risk Communication in Osteoporosis) study, a landmark international survey of 332 postmenopausal women with osteoporosis or at risk of fracture conducted across 11 sites worldwide, provided the most comprehensive evidence to date on the gap between what patients want and what they receive [17]. Although participants rated the importance of receiving fracture risk information very highly (mean 6.2 on a 7-point Likert scale), only 56% reported having actually received such information from their healthcare professional. Almost all participants considered it important to discuss both their fracture risk and the consequences of fractures with their clinician. The RICO study thus demonstrated a significant communication gap between healthcare professionals and patients in the discussion of osteoporosis fracture risk.

A subsequent analysis from the RICO project [18] examined the fracture risk decision point (FRDP), defined as the probability at which patients would be willing to accept a medication prescription. Most patients reported an FRDP that was at or below their nationally recommended treatment threshold, indicating a willingness to accept treatment at even lower risk levels than guidelines mandate. Importantly, numeracy levels significantly influenced the FRDP: patients with higher numeric literacy reported a significantly higher median FRDP (10%) compared with those with lower numeracy (5%, *P* < 0.001). This finding underscores that the way risk is communicated, and patients’ ability to process numerical information, directly influences treatment decision-making.

Further clarity on this issue has been offered by arguing that the fundamental problem is not a “treatment gap” but rather a “care gap” [19]. This perspective emphasizes the importance of providing information on the causes and consequences of osteoporosis during clinical encounters, communicating the benefits and risks of treatment in absolute terms so that patients can understand what treatment will mean for them personally, and using decision aids to assist in these conversations. Moreover, a recent position statement [20] further defines “care gap” by emphasizing that optimal osteoporosis care should extend beyond treatment initiation to include timely assessment, diagnosis, multidisciplinary management, and person-centered support. This perspective highlights the importance of communication, shared decision-making, and care that is understandable, equitable, and aligned with patients’ needs and preferences.

## Research on Risk Communication

### Numerical Probability and its Limitations

The health communication literature has extensively documented the challenges patients face in understanding numerical probabilities. There is a widespread “statistical illiteracy” among both patients and physicians alike, with many individuals unable to correctly interpret percentages, conditional probabilities, or relative risks [21, 22]. Key cognitive barriers include denominator neglect (the tendency to focus on the numerator while ignoring the denominator), ratio bias (the perception that “10 out of 100” is different from “1 out of 10”), and the affect heuristic (the tendency to rely on emotional reactions rather than numerical reasoning when assessing risk) [23].

Physicians presented with conditional probabilities frequently made errors when calculating positive predictive values, yet the same physicians performed dramatically better when the identical information was reframed as natural frequencies [24]. Patients frequently underestimate their actual fracture risk when presented with tools like FRAX, perceiving high-risk scores (e.g., ≥ 20%) as low or intermediate. Similarly, women with morbidities linked to elevated risk often misjudge relative risks compared to peers, showing poor discrimination even when stratified by self-perceived categories like “much higher” vs. “about the same” [25]. Moreover, self-perceived risk correlates weakly with actual fracture incidence, with only ~ 30% of those who fractured viewed their baseline risk as elevated. Even after fractures, patients on osteoporosis medications or supplements often perceive lower risk than calculated, limiting motivation for sustained action [26].

### Natural Frequencies and Icon Arrays

Natural frequencies (statements framed as “X out of 100 people like you”) are consistently easier for people to understand than equivalent information expressed as percentages [21, 24]. Natural frequencies specify a reference class, avoid the abstraction of probability, and align with how human cognition naturally processes proportional information. Even children can perform Bayesian inference when information is presented as natural frequencies, whereas they fail when given conditional probabilities [27].

Icon arrays (pictographs) extend this principle by making the affected and unaffected populations visually concrete. A typical pictograph shows 100 human figures, with a subset highlighted to represent those who will experience the event. Icon arrays improve understanding of proportions, reduce denominator neglect, and produce better-calibrated risk perception across diverse populations, including those with low health literacy [28, 29]. Moreover, supplementing numerical risk information with icon arrays substantially improves risk comprehension, even among individuals with very low numeracy, and that these benefits are maintained over time [30].

### Biological Age as a Communication Frame

An alternative approach to risk communication involves translating probability into a more intuitive frame: biological age. Rather than telling a patient their fracture risk is 15%, the concept of “skeletal age” [9, 31] conveys that their skeleton is behaving like that of someone older—for example, “your skeleton has the fracture risk of an average 75-year-old.” This approach has been most extensively studied in cardiovascular medicine, where “heart age” (also called “vascular age” or “cardiovascular age”) has been implemented in numerous risk communication tools [32–34].

A systematic review found that compared with presenting absolute risk, heart age framing increased both risk perception and negative emotional response, particularly among individuals whose calculated heart age exceeded their chronological age [32]. In clinical settings, heart age combined with counselling improved risk factor control (cholesterol, blood pressure, absolute risk) compared with usual care in 4 of 5 trials [32]. However, the evidence that heart age independently motivates behaviour change beyond what is achieved by absolute risk alone remains limited, and concerns have been raised about the potential for heart age to cause disproportionate anxiety or to inflate risk perception in low-risk individuals [5].

### Framing Effects and the Choice of Metrics

A recent study found that presenting patients with their individualized absolute risk reduction significantly increased treatment acceptance [35]. This finding provides empirical support within osteoporosis for the theoretical argument that risk contextualization (presenting risk alongside what treatment can do about it) enhances treatment decision-making. This further supports the use of absolute rather than relative metrics in patient communication, consistent with broader recommendations in the risk communication literature [22], and with calls for presenting treatment benefits in absolute terms as a means of closing the osteoporosis care gap [19].

Whether improved risk communication actually changes patient behaviour remains a key question. Evidence from cardiovascular medicine suggests that pairing risk information with actionable strategies, such as treatment options and lifestyle recommendations, is more effective than risk information alone [36], a concept known as “actionability” [37]. This finding has direct implications for osteoporosis. A patient informed of a 20% five-year fracture risk, but given no guidance on available treatments, may be left feeling anxious with no clear path forward. By contrast, a patient told that their risk is 20% and that treatment could reduce it to 10% has both a reason to act and a pathway to action. The distinction between alarming patients and empowering them hinges on whether risk information is accompanied by context.

## Three Gaps in Current Osteoporosis Risk Communication

The evidence reviewed in Sects. 2 and 3 reveals three critical gaps in how fracture risk is currently communicated to patients.

### The Consequence Gap

Current fracture risk assessment tools communicate the probability of fracture, but offer no insight into what that fracture would actually mean for the patient. This is a significant omission, the consequences of fracture are precisely the information patients value most, as the RICO study clearly demonstrated [38].

Hip fracture is associated with a 1-year mortality rate of approximately 20–22%, with recent systematic reviews documenting excess mortality (above age-matched controls) ranging from 8.4% to 36% in the first year [39–41]. A meta-analysis found that women had a 5-fold increase and men an 8-fold increase in relative likelihood of death within the first 3 months following hip fracture [41]. This excess mortality persists for years: mortality remains elevated for at least 5–10 years after hip fracture in both sexes [39].

Vertebral fracture dramatically increases the risk of subsequent fracture. Among 377,561 female Medicare beneficiaries who sustained a fracture, 10% had another fracture within 1 year, 18% within 2 years, and 31% within 5 years [42]. The risk of subsequent fracture is highest in the immediate post-fracture period, a concept described as “imminent risk” [43, 44]. Research has demonstrated that following a first fragility fracture, the risk of a subsequent fracture within the first year is five times greater than in women with no prior fracture history [45]. Evidence shows that following an initial fracture, the risk of a major osteoporotic fracture is 2.7 times higher than the population baseline at one year, gradually declining to 1.4 times the population risk at ten years [43].

Beyond mortality and refracture, hip fracture is associated with loss of independence, institutionalization, chronic pain, depression, and reduced quality of life. More than half of hip fracture patients do not regain pre-fracture mobility in the first year [46]. Yet none of this information is communicated by current risk assessment tools. A patient told that their fracture risk is 15% has no information about what that fracture would mean for their survival, their independence, or their likelihood of further fractures. This has been likened to informing a patient of their heart attack risk without disclosing that heart attacks can be fatal — it strips the risk estimate of both its clinical significance and its emotional weight [8].

### The Controllability Gap

Current fracture risk assessment tools present risk in a vacuum. They tell patients what their risk is, but not what can be done about it. A 20% fracture probability over 10 years without information on how treatment would change that probability leaves patients with a number but no actionable path forward.

This matters because risk information without efficacy information may paradoxically increase fatalism rather than motivate treatment. Evidence from health psychology demonstrates that perceived risk alone is a weak predictor of health behaviour; the combination of perceived risk and perceived efficacy (the belief that effective action is available) is far more predictive [37]. In the osteoporosis context, a patient who understands both that their risk is high and that bisphosphonates can reduce that risk by 40–70% has a reason to act and a pathway to action. A patient who understands only that their risk is high may simply feel helpless.

Effective anti-osteoporosis medications exist, including bisphosphonates (alendronate, risedronate, zoledronic acid), denosumab, and anabolic agents (teriparatide, romosozumab), which have been shown in randomized clinical trials to reduce fracture risk by 30–70% [47–50]. Contextualization of fracture risk within treatment options, showing patients how their risk profile would change with treatment, is essential for shared decision-making but is absent from all widely used fracture risk tools.

### The Format Gap

Despite decades of research establishing that numerical probabilities are poorly understood by many patients, fracture risk tools have continued to prioritise statistical accuracy over communication effectiveness. Natural frequencies such as “18 out of 100 women like you will fracture in the next 10 years,” icon arrays, and age-based frames have all been shown to enhance comprehension and personal relevance, yet as reviewed in Sects. 2 and 3, none of these formats are incorporated into any existing risk calculator. The result is a persistent format gap between the evidence on effective risk communication and the tools available to clinicians in everyday practice.

### BONEcheck^™^: Addressing the Three Gaps

BONEcheck™ (bonecheck.org) is a digital tool for personalized bone health assessment developed using data from the Dubbo Osteoporosis Epidemiology Study (Australia) and the Danish Nationwide Registry [9, 31]. Unlike conventional fracture risk tools that report a single fracture probability, BONEcheck was designed to address the three communication gaps identified in this paper. It comprises three modules: data input, risk estimation, and risk contextualization (controllability gap). Users are asked to optionally create an account to allow their responses and risk estimates to be saved and retrieved for future use. The osteogenomic profile field is optional and is not required for risk estimation; most users do not provide this information. Information entered by users is handled according to the BONEcheck privacy policy and is not used by funders or external commercial parties in the development or interpretation of individual risk estimates.

### Communicating Consequences: Mortality and Refracture Risk

Using the Garvan Fracture Risk Calculator, BONEcheck estimates the 5-year probability of any fragility fracture and hip fracture, together with subsequent fracture risk and mortality risk. Mortality was incorporated in the underlying Markov transition model rather than competing risk adjustment. The model allows transitions between health states, including death, to be represented directly. We recognise that communicating mortality risk may be distressing or counterproductive for some patients. The skeletal age concept was developed partly to provide an alternative way of communicating the health burden associated with fracture risk in a more interpretable and patient-centred format.

We thought that a 5-year horizon, in older adults, it is more practical and clinically meaningful than a 10-year time frame. Mortality risk is communicated through *skeletal age*, defined as chronological age plus the estimated years of life lost attributable to fracture or risk factors associated with excess mortality [9, 31]. A skeletal age greater than chronological age indicates that the individual has a risk profile comparable to that of an older person with fewer adverse risk factors.

This approach addresses the consequence gap by extending risk communication beyond fracture probability alone. Rather than reporting only the likelihood of fracture, BONEcheck also quantifies the risk of subsequent fracture and communicates the associated increase in mortality risk. Thus, the emphasis shifts from the possibility of fracture to its broader implications for health and survival. This approach is consistent with findings from the RICO study, in which most participants expressed a desire to be informed about the serious consequences of fracture, including loss of mobility, independence, and quality of life [17].

**Fig. 1:**
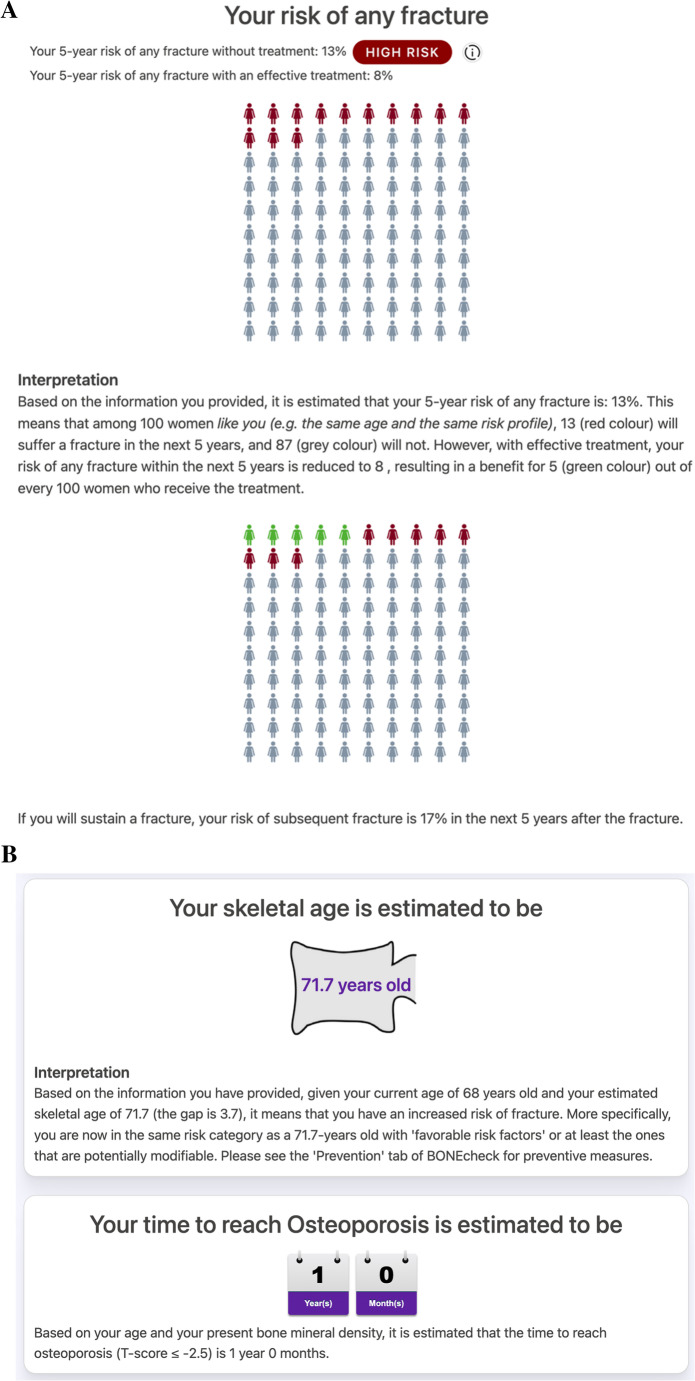
**A **Fracture risk assessment in a 68-year-old woman performed with BONEcheck, presented in pictograph form with contextual information. **B ** Fracture risk assessment in a 68-year-old woman using skeletal age as a metric of risk and the estimated interval for repeat bone mineral density measurement

For example, in a 68-year-old woman with no prior fracture, a femoral neck T-score of − 2.2, two falls in the previous year, and no major comorbidities, a conventional tool might estimate a 5-year fracture risk of approximately 13%. BONEcheck would additionally estimate her skeletal age (e.g., 71.7 years), quantify her 5-year refracture risk if fracture were to occur (17%), and place these estimates in a mortality context (Fig. 1A and B). In this way, the patient is informed not only of her risk, but also of its clinical significance.

### Risk Contextualization: Treatment Benefit Within the Tool

BONEcheck also contextualizes fracture risk by linking it to treatment and management options. The contextualization module presents the potential reduction in fracture risk and survival benefit associated with anti-osteoporosis treatment, based on current Australian guidelines [9]. For example, the tool may indicate: “Your current 5-year fracture risk is 13%. With treatment, this could decrease to approximately 8%.” These estimates are derived from published efficacy data from randomized clinical trials of anti-osteoporosis therapies [47–50].

This approach addresses the context gap by transforming fracture probability from an abstract estimate into clinically actionable information. Presenting baseline risk alongside treatment benefit allows patients to visualize the potential gain from intervention. This is in keeping with evidence from health psychology showing that risk perception is more likely to prompt behaviour change when accompanied by perceived efficacy [37].

BONEcheck further strengthens this contextualization by linking fracture prevention to survival. By showing that treatment may reduce mortality as well as fracture risk, the tool helps patients recognize fracture as a serious health event rather than an isolated outcome.

### Multi-Format Presentation: Probability, Icon Arrays, and Skeletal Age

BONEcheck presents fracture risk in both numerical and icon-array formats (Fig. 1A) [9]. The icon array displays risk within a 100-person grid, making probabilities more tangible and reducing biases associated with percentage-only presentations, such as denominator neglect and ratio bias [28, 29]. The tool also provides skeletal age, offering an age-based representation that may be easier to interpret for individuals with limited numeracy.

This approach addresses the format gap by presenting the same information in three complementary ways: numerical probability for precision, icon arrays for visual comprehension, and skeletal age for personal relevance. These design choices are consistent with the RICO study, which found that patients prefer visual presentation of fracture risk [17], with Trevena and IPDAS recommendations that no single format is universally optimal [51]. They also align with recent guidance supporting the incorporation of visual aids in to fracture risk communication [52]. Furthermore, BONEcheck uses language adapted for individuals with reading proficiency at level 8 or above, thereby helping to reduce health literacy barriers [9].

## Implications for Practice, Research, and Guidelines

### Implications for Clinical Practice

Clinicians should move beyond reporting a single fracture probability. At a minimum, fracture risk should be communicated using natural frequencies or icon arrays, supplemented by information on the consequences of fracture (mortality, refracture risk) and contextualized within treatment options. Skeletal age provides an additional patient-centred frame that may be particularly effective for moderate-risk patients who do not perceive themselves as being at risk. The BONEcheck platform offers a readily available tool that implements these principles, though clinicians should be aware of its current limitations in population calibration.

In clinical consultations, we suggest beginning by establishing (1) the clinical meaning of fracture risk, including the potential consequences of fracture for pain, mobility, independence, and quality of life; (2) the patient’s absolute fracture risk can then be presented using clear numerical and visual formats; followed by (3) discussion of how treatment or other actions may reduce that risk. This three-step approach addresses all three gaps within a single consultation and responds directly to the patient preferences identified in the RICO study [38].

### Implications for Research

Several research priorities emerge from this review. First, randomized controlled studies comparing consequence-inclusive, context-rich, multi-format communication (as implemented in BONEcheck) with conventional single-probability communication on patient outcomes (comprehension, risk perception, treatment uptake, and adherence) are urgently needed. The RICO study provided foundational evidence on patient preferences, but preferences do not necessarily predict behaviour change; intervention studies are required [18, 38]. Future studies should assess not only patient preferences and behavioural outcomes, but also the accuracy with which patients interpret risk information. Visual formats that are preferred by patients may still be problematic if they lead to denominator neglect or misinterpretation of absolute risk. Risk visuals should therefore be evaluated for comprehension, interpretive accuracy, and consistency with established decision-aid standards.

Second, although the Garvan algorithm embedded in BONEcheck has been widely validated in independent populations, the mortality and refracture models incorporated into BONEcheck still require validation in more diverse populations beyond the Australian and Danish cohorts. Third, head-to-head comparisons of risk-communication formats, such as icon arrays, natural frequencies, traffic-light displays, and bar charts, are needed in osteoporosis-specific populations. These studies should assess not only patient preference, but also accuracy of risk interpretation, comprehension, acceptability, and effects on informed decision-making. The RICO study’s finding that patients preferred traffic-light coloured graphs is an important starting point, but comparative effectiveness evidence for different visual formats is lacking [38]. Fourth, studies examining how risk contextualization (treatment benefit framing) influences patient decision-making in osteoporosis are needed, drawing on analogous work in cardiovascular medicine [36].

Fifth, the concept of skeletal age requires further empirical evaluation. The evidence from cardiovascular heart age is more nuanced than is sometimes acknowledged (“heart age” improved risk perception, but provided little evidence of independent behaviour change [32]). Similar careful evaluation is needed for skeletal age in osteoporosis. Sixth, the relationship between numeracy, communication format, and treatment decision-making identified in the FRDP analysis [18] should be explored further to determine whether visual formats can mitigate the influence of numeracy on treatment decisions.

### Implications for Guidelines

Clinical guidelines should incorporate specific recommendations on *how* fracture risk should be communicated, not only at what threshold treatment should be initiated. We recommend that guidelines address: (1) presentation format (recommending natural frequencies, icon arrays, or skeletal age alongside numerical probabilities); (2) consequence disclosure (recommending that mortality and refracture risk be communicated alongside fracture probability); (3) treatment contextualization (recommending that fracture risk be presented alongside expected treatment benefit); and (4) the use of validated digital tools that integrate these principles. These recommendations are overdue and would bring osteoporosis guidelines into alignment with the broader evidence base on risk communication in health.

Patients were not directly involved in selecting all metrics and visual displays included in the current version of BONEcheck. We recognise this as a limitation. Future work should involve patients, clinicians, and decision-aid experts in co-designing and evaluating which statistics are most understandable, acceptable, and useful for treatment decision-making.

A limitation of the current implementation is that BONEcheck is based on the Garvan fracture risk assessment model rather than FRAX, which is used in many clinical guidelines. The Garvan model was selected because the underlying equations are published and freely available, enabling transparent implementation. Future development could examine the feasibility and value of incorporating FRAX or FRAX II estimates, or presenting comparative estimates where this would support clinical interpretation.

## Conclusion

A fracture probability alone is an incomplete piece of information. Patients deserve to know not only their risk of fracture but what that fracture could mean for their life, what they can do about it, and to receive this information in a format they can understand. Previous studies demonstrated, patients want to discuss consequences, they prefer visual formats, and they are willing to act on what they learn [18, 38]. A robust evidence base for improved risk communication already exists, spanning foundational research on natural frequencies and statistical literacy, icon array studies in health decision aids, biological age communication in cardiovascular and respiratory medicine, and osteoporosis-specific findings [18, 38, 52].

Closing the osteoporosis treatment gap requires closing the communication gap. By integrating mortality risk, refracture risk, skeletal age, treatment contextualization, and multi-format presentation into a single platform, it addresses the consequence gap, the context gap, and the format gap that characterize current fracture risk communication. Although the challenge is substantial, there are practical tools and strategies that could help improve risk communication and support better-informed decisions.

## Data Availability

No datasets were generated or analysed during the current study.
